# Effect of Smartphone on Hand Performance and Strength in the Healthy Population

**DOI:** 10.7759/cureus.15798

**Published:** 2021-06-21

**Authors:** Jasraj Kaur Bhamra, Waqar M Naqvi, Sakshi P Arora

**Affiliations:** 1 Community Physiotherapy, Ravi Nair Physiotherapy College, Wardha, IND; 2 Community Physiotherapy, Datta Meghe Institute of Medical Sciences, Wardha, IND

**Keywords:** handfunction, qdash questionnaire, smartphone overuse, smartphone addiction scale (sas-sv), handgrip strength

## Abstract

The study aimed to determine the interactive effects of smartphone use on hand grip strength as well as functional hand performance in young people. The evolution of technology smartphone has become our necessity. It has made our lifestyle more comfortable in the form of browsing the internet, important conversation, and source of entertainment. However, it has negatively impacted our lifestyle too. Smartphone consumption among the young population has become broadly popular for different purposes aside from communication including playing games as well as internet browsing. The main complications associated with the increase in the use of smartphones results in weakness of the hand as well as wrist. High levels of smartphone use diminished hand grip strengths as well as and hand function leading to decreased hand grip strength in their dominant hand.

## Introduction and background

These days, modern innovative technology plays a crucial part in our life. This requires staying aware of continuous advances in communication technologies. Touchscreens became the primary interaction method for smartphones due to their ability to combine input and output in a single interface [1]. Smartphones are no longer just "mobile phones," but also powerful portable computers that give real-time information. Despite the benefits of smartphone usage, such as improved social networking and increased productivity [2], depression, anxiety, accidents, poor sleep, poor academic performance, exhaustion, and high stress have all been linked to this smartphone addiction [3]. Overuse has also been shown to have negative impacts on physical and mental health. Neck pain symptoms are an example of negative bodily impacts [4]. The observation made us believe that the increased time that the young generation is spending on smartphones is making them more addicted to smartphones. Additionally, because of its particular application, social engagement between individuals is compromised by reducing face-to-face connection and encouraging more chat room communication. This may result in a lack of real-life social interaction, which can lead to relationship problems, and interfere with students' academic progress [5]. In adolescents, the rates of smartphone addiction in the high-risk and low-to-medium-risk groups were 2.2 and 9.3% respectively, and 1.0 and 6.7% respectively, in adults [6].

The Cronbach's alpha correlation coefficient of the smartphone addiction scale short version (SAS-SV) is 91 [7]. The Smartphone Addiction Scale (SAS) was the first scale for diagnosing smartphone addiction. This scale comprises 33 components and has been reported to be dependable and consistent internally [8].

Humans have two distinct grips on their hands: power and precision [9]. The ability to determine the efficacy of various treatment techniques or the impacts of various procedures requires a reliable and valid assessment of hand strength [10].

The addiction to smartphones has an impact on the power and precision of hand. The qDASH (Questionnaire for Disabilities of the Arm, Shoulder, and Hand) is widely used to determine the extent of the impact of smartphones on hand performance and strength. This questionnaire is for people with disabilities of the arm, shoulder, and when establishing the efficacy of various treatment options or the impacts of various operations, a reliable and valid assessment of hand strength is critical. It is commonly acknowledged that grip and pinch strength are important [11].

## Review

Smartphones have become a basic component of life in the current setting, for communication as well as vital social extras. A smartphone was demonstrated as a prototype that detects capacitive touch input from all components of the grips of the hand. The hand movement created heat maps by recording the finger positioning in the form of capacitive pictures of common grips that the hand follows characteristically while holding or operating the smartphone [1].

Despite the benefits of smartphone use like improved social networking, higher productivity, and more dynamics along with instant methods of working and living, a previous study reveals that individuals getting overexposed to their phones in different ways compelled them to restrict their everyday life. Smartphone addiction was more prominent among medical college students with different relationships between male and female smartphone usage and their psycho-behavioral variables. These findings point to the need for treatments to help undergraduate students overcome their smartphone addiction [2]. Excessive smartphone use was assessed among 195 medical students which suggested students suffer from a high level of smartphone addiction. It also demonstrated the strong link between this smartphone addiction and lower sleep quality along with higher stress levels, which is a cause for medical concern [3]. Smartphone usage and addiction when assessed in 1,519 young people had provided the first insights into smartphone addiction as well as its use, and smartphones as a medium of addiction in youngsters [4]. By focusing on the impact that the smartphone has created on education, psychology, and social aspects, the good and bad effects of smartphones on students have been identified [5]. This review is critically determining and analyzing if smartphone use has a beneficial or bad impact on students' lives.

A small version of the smartphone addiction scale (SAS-SV) was curated as a computing platform with more and more advanced and specific data processing the addictive requirements as well as the connection. The goal of this study was to create a self-diagnostic scale for internet and smartphone usage characteristics that might detect smartphone addiction. The SAS-SV showed good reliability and validity for the assessment of smartphone addiction [6]. The validity and the reliability of the smartphone addiction scale-short (SAS-SV) were separately assessed and demonstrated for adolescents. The results showed that this scale had strong validity as well as reliability scores, indicating that it may be utilized to assess individuals' levels of smartphone addiction [7]. The psychometric features of the Smartphone Addiction Scale (SAS) featuring internal consistency, dimensionality, and concurrent and construct validity supported the conclusion that SAS is a reliable as well as a valid instrument for testing smartphone addiction [8].

Valid screening scales are available to identify behavioral problems related to this technology, usually in adolescent populations, as research on smartphone addiction has gone along with the scientific literature on problematic mobile phone use developed over the years. The short version of the Smartphone Addiction Scale [SAS-SV] proposed the addiction somatic symptoms did not cover the complete group of estimated excessive users. The scale revealed that no more than 60% of excessive smartphone users approved withdrawal along with tolerance symptoms [12]. The volunteers' understanding together with interpreting the scale described no uncertainties. The results linked with test-retest evaluation give good reliability of the responses given by participants. Regarding construct validity, all the variables associated with time indicators were significantly and positively associated with maximum SAS-SV scores [13].

The psychometric features of the SAS-SV following the goals of the SAS-SV structure were determined by various statistical approaches, indications of convergence, the SAS-SV temporal stability, and SAS-SV predictive validity. These findings suggest that the SAS-SV could be a reliable tool for population research. The instrument also had a moderate connection with smartphone-related factors (number of messages sent as well as received, time spent on smartphones, and number of times looked into a smartphone) making it more precise and accurate [14].

A smartphone has become a necessity as a result of technological improvements [15]. On the one hand, it has made our lives easier by allowing us to browse the internet, have essential conversations, and have a source of pleasure. However, on the other hand, the link between smartphone addiction and grip strength and upper limb disability has been left unnoticed. The use of a smartphone reduces hand grip strength and increases upper-limb disability. Female students were found to have a somewhat greater rate of smartphone addiction [15]. Upper limb impairment was found to be mild to severe, with male students being slightly more affected and had much higher hand grip strength than female students [15]. While assessing the effective interaction of smartphone use along with hand dominance on children's grip strength and functional hand performance, the high levels of smartphone use were found to harm hand grip strength as well as hand function. That is, the grip strength of high-frequency smartphone users' dominant hands was diminished. Both high and low-level smartphone users had problems with hand function in their respective dominant hands [16].

One of the most common consequences related to the increased use of smartphones is hand and wrist weakness [16]. This weakness is caused by repeated flexion and extension of the finger, thumb, and wrist which leads to greater musculoskeletal disorders. The association between smartphone usage duration (as measured by the phone's capacity to track screen time) and hand-grip are incompletely defined. As a result, a link between smartphone usage duration as well as hand-grip strength in young adults was investigated in which the long-term smartphone use was linked to a weaker grip grasp and the length of time spent on a smartphone, as well as age which may play a role in the strength of the hand muscles [17]. The grip strength in healthy young Indians was assessed using data from 1005 healthy volunteers with a mean age of 22 years for both males and females demonstrating that the least age limit was chosen at 18 years to allow for musculoskeletal maturity, while the maximum age limit was set at 30 years to account for the increasing loss of functional independence [9].

The non-dominant hand is 5-10% less functional than the dominant hand [10]. Furthermore, this was not the case for 5-10% of left-handed people, who had a stronger grip strength in their non-dominant hand [18]. It is generally believed that grip strength gives an objective index for the functional integrity of the upper extremity as well a reliable and valid assessment of hand strength which is necessary for regulating the efficacy of treatment measures [10]. The grip strength differences between left and right-handed people suggested that the dominant hand of right-handed people is much stronger than the left hand. However, there was no such significant variation between sides of left-handed people [10]. Grip strength is now widely accepted as the most accurate clinical indicator of human strength. However the average maximum grip strength in all age groups is 10 to 15% lower than peak grip strength [19].

The disabilities of the arm, shoulder, and hand (DASH) questionnaire is a region-specific and self-managed outcome instrument used to quantify upper-extremity impairment along with symptoms. The results prescribed that the Swedish version of the qDASH is viable as well as a reliable tool. It should be a useful instrument in clinical studies of upper-extremity musculoskeletal diseases and prenatal abnormalities [11]. The validity and reliability of the British English version of arm, shoulder, and hand impairments demonstrated how Rasch analysis was used to create a shorter, more rapidly administered version of the DASH. Concurrent and discriminant validity, internal consistency, test-retest reliability, and sensitivities were among the psychometrics evaluated [20].

The advantage of generic measures is that they may be used with a wide range of populations. Making and administering scoring procedures and selection which are easier to accept and implement, and additionally, normative data is frequently likely to help in the interpretation of scores [21]. The DASH questionnaire can be helpful for a range of upper extremity disorders as well as shoulder-specific assessments like the Shoulder Pain questionnaire are examples of this. QuickDASH scores were marginally higher than DASH scores [22].

## Conclusions

In this review, we found that young adults have a significantly higher rate of smartphone addiction. Females were shown to be more addicted to their smartphones. However, the upper-limb disability is more prominent in males. Hand strengths were found to be normal and strong on the dominant-hand side with smartphone use. There is a negative influence on hand function and performance due to the overuse of smartphones. It was also found that there is a non-significant positive connection between smartphone addiction and upper extremity dysfunction.
